# Dupilumab-Related Ocular Surface Disease in Atopic Dermatitis: Risk Stratification, Monitoring, and Persistence-Preserving Management

**DOI:** 10.3390/jcm15041651

**Published:** 2026-02-22

**Authors:** Stefano Bighetti, Luca Bettolini, Carlo Alberto Maronese, Federica Macchi, Zeno Fratton, Vincenzo Maione, Mario Valenti, Giovanni Paolino, Andrea Carugno, Marco Ferrari, Piergiacomo Calzavara-Pinton, Marina Venturini, Nicola Zerbinati, Mariateresa Rossi

**Affiliations:** 1 Dermatology Department, University of Brescia, ASST Spedali Civili di Brescia, 25123 Brescia, Italy; l.bettolini@unibs.it (L.B.); maionevincenzo@gmail.com (V.M.); piergiacomo.calzavarapinton@unibs.it (P.C.-P.); marina.venturini@unibs.it (M.V.); mariateresa.rossi@unibs.it (M.R.); 2 Dermatology Unit, Fondazione IRCCS Ca’ Granda Ospedale Maggiore Policlinico, 20122 Milan, Italy; carlo.maronese@unimi.it (C.A.M.); federica.macchi@unimi.it (F.M.); 3 Department of Pathophysiology and Transplantation, Università Degli Studi di Milano, 20122 Milan, Italy; 4 Institute of Dermatology, Department of Medicine, University of Udine, 33100 Udine, Italy; zenofratton@gmail.com; 5 Dermatology Unit, IRCCS Humanitas Research Hospital, 20089 Milan, Italy; mario.valenti@hunimed.eu; 6 Dermatology Unit, Università Vita-Salute San Raffaele, 20132 Milan, Italy; paolino.giovanni@hsr.it; 7 Dermatology Clinic, IRCCS San Raffaele Scientific Institute, 20132 Milan, Italy; 8 Department of Medicine and Surgery, University of Insubria, 21100 Varese, Italy; andrea.carugno@uninsubria.it; 9 Center for Research in Medical Pharmacology, University of Insubria, 21100 Varese, Italy; marco.ferrari@uninsubria.it; 10 Department of Medicine and Innovation Technology (DiMIT), University of Insubria, 21100 Varese, Italy; nicola.zerbinati@uninsubria.it

**Keywords:** dupilumab, atopic dermatitis, ocular surface disease, conjunctivitis, dupilumab-associated ocular surface disease, drug survival, risk stratification, management

## Abstract

**Background/Objectives**: Dupilumab-related ocular surface disease (DROSD) is a significant safety challenge in atopic dermatitis (AD) management, potentially leading to treatment interruption despite cutaneous efficacy. This narrative review evaluates risk stratification and management strategies to standardize monitoring and preserve long-term drug persistence. **Methods**: A search of PubMed/MEDLINE was conducted from inception to 31 December 2025. Evidence was synthesized from clinical trials, pooled safety analyses, and real-world registries, focusing on risk factors, monitoring tools, and interdisciplinary management algorithms for DROSD in AD populations. **Results**: Clinical trials identify conjunctivitis as a reproducible, context-dependent signal enriched in AD populations. Real-world data highlight that ocular symptoms disproportionately drive treatment dissatisfaction and discontinuation. Clinical vigilance must extend throughout the treatment course; while many cases appear early, a significant proportion develops between 8–16 weeks, with late-onset manifestations reported up to 12 months after initiation. Effective management relies on baseline risk documentation—including prior ocular history and AD phenotype—and the implementation of stepwise, severity-based “treat-through” protocols. **Conclusions**: Managing DROSD is a critical strategy for maintaining treatment persistence. Integration of routine baseline risk capture, continuous symptom surveillance, and structured multidisciplinary escalation pathways is essential to maximize ocular safety and long-term therapeutic outcomes in AD.

## 1. Introduction

Atopic dermatitis (AD) is a chronic inflammatory disease wherein long-term control increasingly relies on targeted systemic therapies. Parallel to this therapeutic shift, dupilumab-related ocular surface disease (DROSD)—also referred to as dupilumab-associated ocular surface disease (DAOSD) or dupilumab-induced ocular surface disease (DIOSD)—has emerged as a practice-relevant safety domain. Consequently, recent interdisciplinary guidance has proposed structured, severity-based pathways to support standardized monitoring and escalation in routine care [1,2,3,4].

Systematic syntheses and ophthalmology-anchored cohorts suggest that ocular surface manifestations may persist or recur in a subset of patients and, in selected cases, may be associated with longer-term sequelae, reinforcing the importance of early recognition and timely escalation when indicated [4,5,6].

Dupilumab remains a cornerstone treatment for moderate-to-severe AD, supported by robust efficacy and a generally favourable safety profile in pivotal phase 3 trials and long-term extension studies [7,8,9]. However, clinical development programmes consistently identified conjunctivitis and related ocular events as a reproducible signal in dupilumab-treated AD populations. Pooled analyses indicate that this signal is enriched in AD compared with other dupilumab-treated indications, supporting a context-dependent phenomenon occurring in an AD-vulnerable ocular surface rather than a uniform toxicity across diseases [10].

In routine practice, the effectiveness and safety of dupilumab have been confirmed by large real-world registries and multicentre cohorts [11,12,13,14]. These datasets emphasize a clinically relevant reality: treatment persistence (drug survival) is frequently shaped by pragmatic determinants, including adverse events. Within this framework, ocular events are rarely trivial. Conjunctival hyperaemia, tearing, irritation, foreign-body sensation, photophobia, and blepharitis-/meibomian gland dysfunction–like features may rapidly become the dominant complaint, erode quality of life, increase healthcare utilization, and precipitate treatment interruption even when cutaneous outcomes are excellent [1,2,4,5,6]. Furthermore, emerging real-world evidence suggests that host-related factors—including baseline ocular predisposition—may influence persistence. A recent cohort analysis reported an association between conjunctivitis predisposition (and male sex) and reduced dupilumab drug survival, strengthening the rationale for pre-treatment ocular risk documentation [15].

A further complexity is that AD itself is associated with baseline ocular surface vulnerability, including dry eye symptoms, MGD, chronic conjunctivitis, and atopic keratoconjunctivitis (AKC), which complicates the attribution between disease-related and treatment-associated inflammation [6,16]. Accordingly, a strictly aetiologic distinction is often less actionable than a severity-based approach prioritizing early detection, exclusion of ‘red flags’ (notably corneal involvement), and timely escalation within shared care [1,2,6,16]. In this context, adopting a shared language and operational framework is essential; widely used ocular surface standards such as TFOS DEWS II provide harmonized definitions and stepwise management principles for ocular surface dysfunction that can be adapted pragmatically within dermatology-led care pathways [17,18]. Finally, contemporary endotype-based models of AD underscore heterogeneity in barrier dysfunction and immune polarization that may plausibly modulate ocular susceptibility under IL-4/IL-13 pathway blockade, supporting a risk-informed, phenotype-aware clinical approach [19].

In this narrative review, we synthesize trial evidence, real-world data, and interdisciplinary guidance to provide a pragmatic, clinic-oriented approach to DROSD in AD. Specifically, we address which baseline features should be routinely captured to stratify ocular risk before dupilumab initiation, how to monitor and grade ocular symptoms using feasible tools in high-volume dermatology settings, and how to manage DROSD stepwise—defining thresholds for treat-through, referral, temporary interruption, and switching—to maximize both ocular safety and long-term dupilumab persistence.

## 2. Materials and Methods

This narrative review was conceived to support risk-aware, clinically implementable pathways for dupilumab-related ocular surface disease in atopic dermatitis. The synthesis was developed according to narrative-review best practices, including a clear clinical rationale, transparent description of evidence identification, and balanced interpretation across heterogeneous study designs.

A literature search was performed in PubMed/MEDLINE from database inception to 31 December 2025. Searches combined free-text keywords and MeSH terms covering exposure and target condition (dupilumab; atopic dermatitis/atopic eczema), ocular outcomes and ocular surface disorders (ocular surface disease; conjunctivitis; keratitis; blepharitis; meibomian gland dysfunction; dry eye; atopic keratoconjunctivitis; DAOSD/DIOSD/DROSD), and implementation-relevant domains such as risk factors, screening, prevention, monitoring tools, management algorithms, referral, drug survival, and real-world registries. Searches were iteratively refined to ensure capture of pivotal trials and pooled safety analyses, observational cohorts describing clinical presentation, management and sequelae, systematic reviews/meta-analyses, and consensus or guidance documents.

Additional records were identified by manual screening of reference lists from included articles and relevant reviews, and by targeted retrieval of key documents used to standardize ocular surface terminology and stepwise management principles, as well as AD systemic-therapy guidelines relevant to therapeutic positioning and switching.

Because ocular outcomes are variably defined, ophthalmologic confirmation is inconsistent, follow-up durations differ, and the evidence base spans both trial-level and real-world designs, we did not undertake formal systematic screening, quantitative pooling, or risk-of-bias assessment. Instead, evidence was prioritized for clinical utility and relevance to real-world decision-making, with interpretive caution for retrospective designs and for studies relying on administrative coding.

Where multiple terms were used to describe overlapping entities (DROSD/DAOSD/DIOSD), we treated these as convergent descriptors of dupilumab-associated ocular surface manifestations in AD and mapped study definitions to a pragmatic, severity-oriented framework rather than assuming strict nosological equivalence across publications. Mechanistic or translational observations were incorporated only when they plausibly supported observed clinical patterns or management rationale, and they were interpreted as supportive rather than definitive causal evidence.

Overall, the synthesis was structured around practical clinical tasks—baseline risk documentation, early detection, feasible symptom monitoring in high-volume settings, severity-based stepwise management, referral timing, and persistence-preserving decision-making—rather than a comprehensive mechanistic taxonomy of ocular disease under IL-4/IL-13 pathway blockade.

## 3. Results

### 3.1. Clinical Presentation and Disease Burden

**Figure 1:**
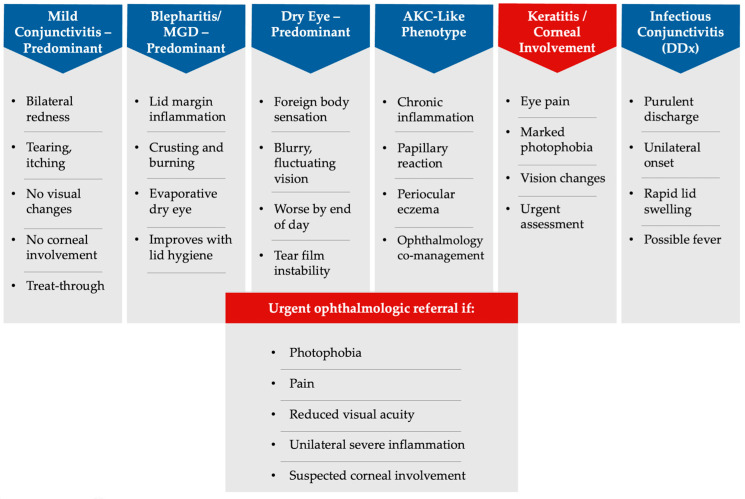
Clinical spectrum of dupilumab-related ocular surface disease (DROSD) in AD. Phenotypes range from conjunctivitis- and blepharitis-predominant forms to AKC-like and corneal involvement; red-flag features and infectious differentials are indicated.

Across dupilumab-treated AD populations, DROSD spans a continuum from mild conjunctival hyperaemia, tearing, and irritation to complex phenotypes with blepharitis/MGD, keratitis, and features overlapping with AKC [1,2,3,4,6,20]. This spectrum is clinically consequential because ocular symptoms can rapidly become the dominant complaint despite excellent cutaneous control, driving dissatisfaction, additional visits, and treatment interruption [1,2,4,5,6]. Interdisciplinary consensus statements have emphasized that these events should be approached as predictable, actionable complications warranting standardized monitoring rather than reactive symptom control [1,2]. Ophthalmology-anchored cohorts further underscore that ocular manifestations may persist or recur and, in a subset, may be associated with longer-term sequelae—particularly when inflammatory activity is prolonged or when corneal involvement is present [6,21,22]. Importantly, multiple observational series indicate that many cases are manageable without discontinuing dupilumab when recognized early and treated systematically, including with topical anti-inflammatory strategies in selected phenotypes [20,21,22,23,24]. These presentations define conjunctivitis-, blepharitis/MGD-, dry eye–, AKC-like, and keratitis-predominant patterns (Figure 1). The practical implication is that DROSD occurs with sufficient frequency and impact to justify its integration into routine systemic-therapy workflows as a persistence-preserving safety domain [1,2,3,4,6].

### 3.2. Evidence from Clinical Trials and Pooled Analyses

The ocular signal is not confined to real-world cohorts. In pivotal phase 3 trials and long-term randomized studies, conjunctivitis and related ocular events were repeatedly reported [7,8,9]. Pooled analyses characterize this signal as enriched in AD compared with other dupilumab-treated indications, suggesting a context-dependent phenomenon in an AD-vulnerable ocular surface rather than uniform drug toxicity [10]. From an implementation standpoint, these datasets justify proactive counselling: ocular events should be framed as anticipated and monitorable, enabling structured pathways to minimize morbidity [1,2,3,10]. Adolescent trial programmes also report conjunctivitis, reinforcing that ocular monitoring should not be relaxed purely on the basis of age, even though outcome definitions and ophthalmologic confirmation vary across studies [25]. Overall, the consistency of trial reporting supports a standardized approach to screening, early detection, and stepwise management in routine care [1,2,3,7,8,9,10].

### 3.3. Pathophysiology and Mechanistic Hypotheses

While a unified mechanism remains unproven, converging evidence supports a context-dependent model in which dupilumab interacts with a type-2–primed ocular surface in patients with AD. AD is characterized by IL-4- and IL-13–driven immune polarization, contributing to epithelial barrier dysfunction, altered antimicrobial responses, and chronic allergic inflammation [19]. Ocular surface homeostasis is tightly regulated by immune–epithelial interactions, and type-2 cytokines contribute to mucosal barrier integrity and goblet cell biology. Importantly, the ocular surface shares structural and immunologic features with the skin, including epithelial tight junction regulation and mucin-dependent tear film stability.

Patients with AD frequently exhibit baseline ocular surface vulnerability—such as dry eye symptoms, blepharitis/meibomian gland dysfunction (MGD), and atopic keratoconjunctivitis (AKC)—reflecting this systemic type-2 inflammatory background [16]. In this context, IL-4Rα blockade may interact with a pre-existing type-2–primed conjunctival microenvironment rather than uniformly suppress inflammation, potentially contributing to the higher incidence of ocular surface disease observed in dupilumab-treated AD populations compared with non-AD indications.

Although IL-4/IL-13 inhibition improves cutaneous inflammation, the ocular microenvironment in AD is not defined solely by active type-2 signaling but also by chronic epithelial fragility, altered barrier integrity, and baseline mucosal instability [16,19]. Pathway inhibition may therefore modify immune–epithelial equilibrium rather than simply extinguish inflammatory activity, offering a mechanistic explanation for the coexistence of cutaneous improvement and ocular surface manifestations. Translational data documenting conjunctival goblet cell scarcity and inflammation during dupilumab therapy support the hypothesis that IL-4/IL-13 pathway modulation may influence mucin production and tear film homeostasis in predisposed individuals [26]. Alterations in mucin-dependent tear film stability could promote epithelial stress and secondary inflammatory activation. In parallel, real-world immunologic observations—such as transient increases in circulating basophils and eosinophils in association with dupilumab-associated conjunctivitis—suggest immune re-patterning rather than direct drug toxicity [27]. Rather than representing a uniform exacerbation of allergic inflammation, DROSD may therefore reflect immune recalibration within a predisposed conjunctival niche [20,26].

Crucially, it remains essential to distinguish between pre-existing ocular morbidity and treatment-associated inflammation. AD itself confers baseline ocular surface fragility; DROSD may therefore represent an inflammatory overlay emerging under pathway modulation rather than a de novo toxic effect. From a clinical perspective, this reinforces the importance of severity-based assessment, early recognition of corneal involvement, and timely shared dermatology–ophthalmology management, rather than strict etiologic categorization alone [1,2,16].

### 3.4. Risk Stratification and Pre-Treatment Assessment

Preserving long-term persistence depends on identifying higher-risk patients before ocular morbidity develops. Early clinical studies identified risk factors for dupilumab-associated conjunctivitis, establishing a practical precedent for structured baseline ocular risk documentation in AD populations [5]. Clinical vigilance must be maintained throughout the treatment course, as the onset of DROSD exhibits a wide temporal range. While some cases emerge within the first weeks of therapy, a substantial proportion develop ocular symptoms between 8 and 16 weeks, and late-onset manifestations have been reported after 6–12 months of treatment [26]. This temporal variability underscores the need for continuous symptom surveillance beyond the induction phase. In routine dermatology practice, several baseline features are feasible and clinically informative to document prior to dupilumab initiation: history of recurrent conjunctivitis or allergic eye disease, symptoms consistent with dry eye, blepharitis/MGD, contact lens use, periocular eczema or AKC, and prior ophthalmology follow-up [1,2,6,16]. These variables do not establish causality but support pragmatic risk awareness and triage planning.

Age may plausibly modulate ocular surface vulnerability, as conditions such as dry eye disease and MGD increase in prevalence with advancing age. However, available data from clinical trials and real-world cohorts do not consistently identify age as an independent dominant predictor of DROSD, with ocular events reported across adult and adolescent AD populations [7,8,9,10,25]. Current evidence therefore suggests that baseline inflammatory phenotype and pre-existing ocular morbidity are more relevant determinants than chronological age alone.

Phenotype context may further inform monitoring intensity. Head-and-neck–dominant AD has been associated with suboptimal disease control in real-world cohorts; although this does not demonstrate direct causation of DROSD, it may function as an operational flag for intensified periocular assessment and closer early follow-up [28,29]. Large database analyses provide complementary context by describing incidence patterns of bacterial and nonbacterial conjunctivitis in dupilumab-treated patients with AD, reinforcing the importance of differentiating infectious from inflammatory events and of systematic outcome capture in registries [30].

From a practical standpoint, dermatologists should be able to identify ocular features that warrant prompt ophthalmologic referral during dupilumab therapy. Referral-trigger criteria include ocular pain, marked photophobia, blurred or reduced visual acuity, unilateral or rapidly progressive inflammation, purulent discharge, significant lid oedema, and any suspicion of corneal involvement [1,2,6,16]. These features may indicate keratitis or severe inflammatory disease and require timely ophthalmologic evaluation irrespective of cutaneous response. In contrast, mild bilateral conjunctival hyperaemia or irritation without visual disturbance may initially be managed within structured treat-through pathways, with escalation guided by symptom persistence or recurrence.

Finally, feasibility-oriented studies suggest that brief patient-reported symptom tools can facilitate early detection in high-volume dermatology settings where detailed slit-lamp examination is not available [31]. While these instruments do not replace ophthalmologic assessment when keratitis or AKC is suspected, they may operationalize earlier recognition—an essential prerequisite for persistence-preserving management. A minimal dermatology-feasible baseline dataset and monitoring toolkit summarizing these domains is provided in Table 1. This framework is designed as a scalable checklist adaptable to different healthcare settings, according to local ophthalmology access and resource availability.

### 3.5. Stepwise Management and Prophylaxis Strategies

**Figure 2:**
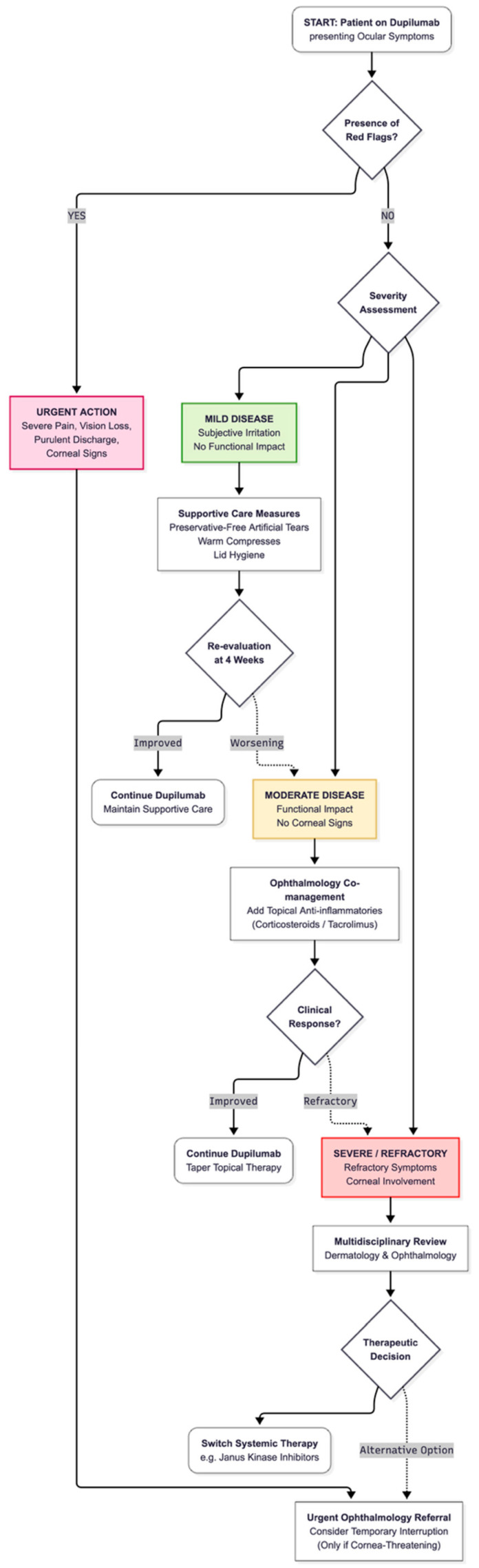
Stepwise, severity-based management pathway for DROSD in AD. JAK-i: Janus kinase inhibitors.

Interdisciplinary guidance advocates for a ‘treat-through’ paradigm: the majority of DROSD cases can be managed without stopping dupilumab when symptoms are detected early and treatment is escalated according to severity [1,2]. For pragmatic implementation, severity may be operationalized by symptom persistence/recurrence and functional impact, with red flags (pain, marked photophobia, vision change, or suspected corneal involvement) defining urgent referral regardless of symptom duration [1,2,6,16,32]. Dermatology–ophthalmology collaborative algorithms align with this approach, emphasizing initial supportive care (lubrication, lid hygiene) and prompt anti-inflammatory escalation when symptoms persist, recur, or worsen [22,24,32]. Real-world experience supports prophylaxis-oriented approaches in selected high-risk phenotypes, and observational studies confirm that treat-through strategies with appropriate topical therapy (including calcineurin inhibitors) often lead to acceptable outcomes [20,21,22,23,33]. Available data further indicate that temporary discontinuation of dupilumab in severe or refractory cases is frequently followed by improvement in ocular symptoms, although resolution may not be immediate and appears to depend on baseline phenotype and the presence of corneal involvement [6,21,22]. Prolonged inflammatory activity, particularly when corneal structures are affected, may be associated with persistent symptoms or structural sequelae. In selected refractory cases, therapeutic switching has been described in real-world practice; emerging evidence suggests that transition to alternative systemic agents, including Janus kinase inhibitors, may be associated with improvement of ocular manifestations in some individuals, although comparative data remain limited. To harmonize language and escalation logic, we suggest adapting established ocular surface frameworks (e.g., TFOS DEWS II) as a conceptual scaffold [17,18]. While DROSD is not identical to dry eye disease, these standards can support consistent terminology and structured follow-up within dermatology-led pathways—especially where ophthalmology access is constrained and triage needs to be explicit [17,18]. A stepwise, severity-based management pathway aligned with interdisciplinary guidance is provided in Figure 2.

### 3.6. Drug Survival and the Real-World Consequence of Ocular Adverse Events

The overarching clinical objective is durable AD control with sustained persistence of effective systemic therapy. Real-world registries and multicentre cohorts demonstrate that dupilumab can maintain long-term effectiveness, but discontinuation occurs and adverse events contribute to reduced persistence [11,12,13,14,34]. Within this landscape, ocular events can be disproportionately disruptive: even when cutaneous outcomes are strong, ocular symptoms may dominate the patient experience and trigger interruption if not recognized and managed early [1,2,6]. Notably, real-world cohort analyses have linked ocular predisposition with reduced dupilumab drug survival [15]. Conceptually, DROSD management is therefore not only symptom control but also a persistence strategy: structured early detection and treat-through pathways may reduce unnecessary interruptions and downstream discontinuation [1,2,15].

### 3.7. Therapeutic Positioning and Switching Considerations

As systemic options for AD expand, safety trade-offs become increasingly individualized. European guideline frameworks position dupilumab alongside other systemic options and support structured decision-making for sequencing and switching [35]. Head-to-head comparative trials show that JAK inhibitors can provide rapid improvements versus dupilumab, but they carry distinct safety considerations (including infection risk and laboratory monitoring) that must be weighed against ocular adverse-event burden when switching is considered [36]. IL-13–targeted biologics have generated substantial phase 3 evidence and integrated safety analyses, including conjunctivitis reporting across trials, underscoring that ocular events are not exclusive to dupilumab and that ocular monitoring may remain relevant across type 2 biologic classes, albeit with differences in rates, definitions, and study populations [37,38,39,40]. In practice, management should prioritize treat-through strategies when safe, while recognizing that interruption or switching may be appropriate in refractory or recurrent severe disease [1,2,35,36,37,38,39,40]. Case-level reports illustrate diagnostic ambiguity between DROSD and baseline AKC, supporting individualized escalation or therapeutic reconsideration when corneal risk remains despite optimized topical therapy [41].

## 4. Conclusions

DROSD has emerged as a principal safety concern in AD, owing to its prevalence, likelihood of recurrence, and ability to diminish quality of life and adherence to treatment in otherwise well-managed patients. Clinical trial programmes and pooled analyses affirm conjunctivitis and associated ocular events as consistent signals in dupilumab-treated AD populations, thereby justifying proactive patient counselling and structured monitoring protocols. Existing interdisciplinary frameworks endorse severity-based, treat-through approaches for numerous cases, complemented by early ophthalmology co-management and explicitly defined referral criteria when keratitis or AKC is suspected. A practical priority involves the implementation of routine baseline risk documentation, feasible symptom surveillance in high-volume dermatology settings, and stepwise escalation strategies that maintain dupilumab persistence whenever deemed safe.

## Figures and Tables

**Table 1:** Dermatology-feasible baseline ocular risk documentation and monitoring toolkit before and during dupilumab for atopic dermatitis.

Domain	What to Capture/do (Dermatology-Led, Feasible)	Rationale/Notes
**Baseline ocular history**	Recurrent conjunctivitis; allergic eye disease; dry eye symptoms/diagnosis; blepharitis/MGD; history/diagnosis suggestive of AKC; prior keratitis/corneal disease; prior ophthalmology follow-up [1,2,6,16,32].	Baseline ocular comorbidity and DROSD phenotypes are frequently intertwined; shared-care frameworks emphasize pre-treatment documentation [1,2,6,16,32].
**Exposures/behaviours**	Contact lens use; prior ocular surgery; chronic topical eye drops.	Pragmatic risk documentation to support triage and referral decisions (practice-based).
**AD phenotype flags**	Periocular eczema; head-and-neck–dominant disease (as a pragmatic flag for closer periocular assessment) [29].	Head-and-neck phenotype may signal difficult disease control; use as operational flag with cautious interpretation [29].
**Rapid symptom screen (≤30 s)**	Irritation/itch; tearing; foreign-body sensation; photophobia; vision change (yes/no) [1,2,17,18,32].	Symptom-based monitoring and escalation logic are central to consensus/pathway approaches; DEWS II supports symptom-led staging concepts [1,2,17,18,32].
**Monitoring schedule**	Symptom screen at each visit for the duration of therapy [1,2,32].	Vigilance is required beyond induction; symptoms typically peak between 8–16 weeks, but late-onset cases can occur after 6–12 months [23,26,30].
**Patient-reported tool (optional)**	Brief questionnaire/symptom prompt to trigger early intervention/referral when ocular exam capacity is limited [31].	Direct evidence on feasibility of patient-reported detection tools in dupilumab-treated cohorts [31].
**Red flags (urgent ophthalmology management)**	Pain; marked photophobia; vision change; suspected corneal involvement; unilateral severe presentation; purulent discharge [1,2,6,17,18,32].	Corneal involvement/sequelae and escalation/triage principles are supported by ophthalmology-anchored cohorts [6,17,18] and interdisciplinary pathways [1,2,32].

## Data Availability

No new data were created or analyzed in this study. Data sharing is not applicable to this article.

## Abbreviations

The following abbreviations are used in this manuscript:

AD	Atopic Dermatitis
AKC	Atopic Keratoconjunctivitis
DAOSD	Dupilumab-Associated Ocular Surface Disease
DIOSD	Dupilumab-Induced Ocular Surface Disease
DROSD	Dupilumab-Related Ocular Surface Disease
IL	Interleukin
JAK-i	Janus Kinase inhibitors
MGD	Meibomian Gland Dysfunction
